# Associating life stages and sexes of Nearctic *Polycentropus* Curtis, 1835 (Trichoptera: Polycentropodidae) using mitochondrial DNA barcoding

**DOI:** 10.1002/ece3.8741

**Published:** 2022-03-24

**Authors:** Alexander B. Orfinger, John C. Morse, Raymond L. Hix

**Affiliations:** ^1^ 7822 Center for Water Resources Florida A&M University Tallahassee Florida USA; ^2^ 3463 Department of Entomology and Nematology University of Florida Gainesville Florida USA; ^3^ 2545 Department of Plant and Environmental Sciences Clemson University Clemson South Carolina USA; ^4^ 7822 Center for Biological Control Florida A&M University Tallahassee Florida USA

**Keywords:** Caddisfly, Endopterygota, female, larvae, taxonomy

## Abstract

Alpha taxonomy of caddisflies (order Trichoptera) is based primarily on male genital morphology. As such, associations of adult females and other life stages typically require conclusive association with the species’ identifiable male. The aim of this study was to use molecular methods to associate females and larvae of *Polycentropus* species represented in the Nearctic. Analysis of mtCOI sequences using distance‐ and tree‐based methods resulted in the association of larvae for 14 species of *Polycentropus* (*P. alabamensis* Hamilton, Harris & Lago, 1990, *P. blicklei* Ross & Yamamoto 1965, *P. carlsoni* Morse 1971, *P. carolinensis* Banks 1905, *P. colei* Ross 1941, *P. confusus* Hagen 1861, *P. denningi* Smith 1962, *P. elarus* Ross 1944, *P. gertschi* Denning 1950, *Polycentropus halidus* Milne 1936, *P. maculatus* Banks 1908, *P. pentus* Ross 1941, *P. rickeri* Yamamoto 1966, and *P. variegatus* Banks 1900) and females for 2 species (*P. carolinensis* and *P. chelatus* Ross & Yamamoto 1965). Searches for, and descriptions of, diagnostic morphological characters for these previously unidentifiable life forms are now possible. The identity of the larva of *P. centralis* Banks, 1914 is confirmed and some interesting phylogenetic relationships and a possible cryptic species and potential synonyms are implied in the results. Targets for future immature‐ and female–male associations are discussed along with a preliminary assessment of morphological differences among larvae.

## Graphical abstract

Caddisfly taxonomy is conventionally based on males, resulting in a relatively poor understanding of females and immature stages. Using mtDNA barcoding, we were able to associate additional females and larvae of Nearctic members of the genus *Polycentropus*. With the new knowledge provided here, descriptive taxonomic studies of the newly associated larvae and females can now be undertaken.

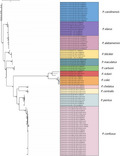

## 1 INTRODUCTION

A key challenge in animal taxonomy is associating morphologically distinct life stages and sexes within a species. This is particularly true of the Endopterygota (= Holometabola), 11 orders of insect that exhibit complete metamorphosis. The immature stages of holometabolous insects are generally markedly different in both ecology and morphology from adults.

The caddisflies (order Trichoptera) are a prime example of a holometabolous order for which our knowledge of nonmales is relatively poor. Among the caddisflies, the taxonomy and identification tools used for aquatic larvae and terrestrial females for most species lag behind those of terrestrial males, on which alpha taxonomy is mostly based. The inability to identify larvae and females limits detailed study of these organisms’ ecology, evolution, and water quality tolerance for development of biomonitoring indices.

There are some notable exceptions to this pattern, however. For example, larvae of the central European caddisfly fauna have been well characterized (Waringer & Graf, 2011). Still, in many regions and for most taxa, male taxonomic knowledge far surpasses that of females, larvae, and eggs. Our taxonomic understanding of the Oriental and Neotropical Trichoptera faunas typify this generality, with immature and female knowledge poorly resolved relative to male taxonomy (Morse, 2016; Pes et al., 2018, respectively).

The Nearctic caddisfly fauna also exemplifies the disparity of male and nonmale caddisfly knowledge (Ruiter et al., 2013). One example of such a taxonomic knowledge gap exists among Nearctic species of the cosmopolitan genus *Polycentropus* Curtis, 1835 (Trichoptera: Polycentropodidae). The genus *Polycentropus* is represented by 30 species in the Nearctic (Rasmussen & Morse, 2020). Larvae of *Polycentropus* construct bag‐like silken filter nets in clean, flowing water with which they capture various small invertebrate prey (Wiggins, 1996). The Nearctic *Polycentropus* fauna can be divided according to geographic distribution, with 23 eastern species and seven western species. This distributional scheme corresponds to the east Nearctic and west Nearctic recognized Trichoptera biogeographical regions (de Moor & Ivanov, 2008) and is reflected in the Nearctic *Polycentropus* species and Species Group distributions (e.g., Hamilton, 1986). The 30 Nearctic *Polycentropus* species are assigned to four monophyletic Species Groups based on synapomorphies of male genital characters (Armitage & Hamilton, 1990; Hamilton, 1986). These include the *Polycentropus arizonensis* Species Group (1 Nearctic species, western), the *P. confusus* Species Group (19 Nearctic species, eastern), the *P. colei* Species Group (3 Nearctic species, eastern), and the *P. gertschi* Species Group (4 species in the Nearctic, western) (Armitage & Hamilton, 1990; Hamilton, 1986). Two additional western species, *P. denningi* Smith, 1962 and *P. variegatus* Banks, 1900 are unplaced (Armitage & Hamilton, 1990). One additional unplaced eastern species, *P. timesis* Denning 1948, is currently assigned to *Polycentropus* but merits redescription and transfer to *Holocentropus* McLachlan, 1878.

Of the 30 species, the larva of only one species (3.3%) and females of only 15 species (50%) have been described. While larval–adult caddisfly associations have traditionally been achieved by rearing larvae or employing the metamorphotype method (*sensu* Milne, 1938), larval and pupal Nearctic *Polycentropus* are morphologically similar, difficult to sample due to their cryptic nature, and have not yet been successfully reared. Female associations are conventionally based on reared individuals, geographic associations, or individuals sampled *in copula* with identifiable males. Modern molecular techniques offer an alternative solution in the form of DNA barcoding.

DNA barcoding employs the mitochondrial cytochrome c oxidase subunit I (mtCOI) fragment of 658 base pairs to identify species in light of the sequence's low intraspecific variability and high interspecific divergence, or barcoding gap, allowing for high success of animal species delineation (Hebert et al., 2003; Ruiter et al., 2013). DNA barcoding has been suggested as an option for associating the different trichopteran life stages and sexes (Barcelos‐Silva et al., 2018; Ruiter et al., 2013; Zhou et al., 2007), as well of those of other aquatic insects including stoneflies (e.g., Mynott, 2015; Mynott et al., 2011) and mayflies (e.g., Malakauskas & Zonca, 2018; Molina et al., 2017). The technique has been used successfully to associate larvae and adults in multiple cases, including in a large variety of caddisfly taxa in North America and Asia (Barcelos‐Silva et al., 2018; Ruiter et al., 2013; Stroil et al., 2018; Zhou et al., 2007). In fact, barcoding exhibits high sequencing success and >95% success in species assignment, including for Trichoptera (Hajibabaei & McKenna, 2012). Even shorter fragments of the COI barcode region of at least 200 bp can reliably identify species in 95% of cases across a variety of taxa (Meusnier et al., 2008; Yeo et al., 2020).

Thanks in part to the Trichoptera Barcode of Life (TBOL) campaign, a robust framework and reference library exist for sequencing, sourcing, and analyzing caddisfly barcoding data (Frandsen et al., 2016; Zhou et al., 2016). By employing DNA barcoding analyses on a wide geographical and morphological variety of larvae and adult males and females, the present study aims to assign species identities to currently unidentifiable larvae and females of the genus *Polycentropus* in the Nearctic. In doing so, this work informs the search for diagnostic morphological characters of larval and female *Polycentropus* species, ultimately making their visual identification possible.

## 2 METHODS

### 2.1 Specimen material

Specimens of Trichoptera housed in the Canadian Centre for DNA Barcoding, Biodiversity Institute of Ontario, University of Guelph, Canada (CCDB), the Clemson University Arthropod Collection (CUAC), the portion of the Florida State Collection of Arthropods (FSCA) housed at Florida A&M University (FAMU), in the US National Park Service (NPS) network of collections, the Illinois Natural History Survey (INHS), the Monte L. Bean Life Science Museum at Brigham Young University (BYU), privately donated material, and material newly collected for this study were used. Late‐instar larvae and adult females were sorted into unique morphotypes. A leg from each of up to 10 specimens of each morphotype was subsampled for DNA. Adult males of each species were also sequenced, or their barcoding sequences sourced from the Barcode of Life Database (BOLD). Finally, available sequences from females, larvae, and pupae identified to genus or species (in the cases of some females) were mined from BOLD. Each of the male specimens whose sequences were mined from BOLD has been identified by a taxonomic expert, including the first author in most cases, and vouchered in a public natural history collection. Each of the female and immature specimens whose sequences were mined from BOLD have been identified by a taxonomic authority, including the first author in many cases, or by Barcode Index Number (BIN) matching *sensu* Ratnasingham and Hebert (2013). Species for which no unknown larval or unknown female sequences of at least 300 base pairs were available were excluded.

### 2.2 DNA extraction and sequencing

DNA amplification and alignment generally follow procedures used by Zhou et al. (2007), Baird et al. (2011), Ruiter et al. (2013), and Barcelos‐Silva et al. (2018). One leg was subsampled from each specimen, and molecular methods follow standard DNA barcoding protocols (Ivanova et al., 2006). DNA extraction and sequencing were accomplished at the Canadian Centre for DNA Barcoding, Biodiversity Institute of Ontario, University of Guelph, Canada (CCDB). DNA was extracted using an AcroPrep 96‐well 3.0‐μm glass‐fiber plate and eluted with 50 μl of distilled water. Extracted DNA was then amplified targeting the full 658‐bp barcoding fragment of COI using polymerase chain reaction DNA amplification and alignment (PCR) in a 12.5 μl reaction volume following the protocol of Ivanova et al. (2006). The reaction was comprised of 6.25 μl of 10% trehalose (D‐(+)‐trehalose dehydrate) (per CCDB standard protocols), 2 μl of ddH_2_O, 1.25 μl 10x of reaction buffer, 0.625 μl 50 mM MgC_l2_, 0.0625 μl of 10 mM dNTP, 0.06 μl of 5 U/μl Taq DNA polymerase (Invitrogen), 0.125 μl of 10 μM of both forward and reverse primer, and 2 μl of DNA. The primer used to amplify the full barcoding region was (LCO1490 50‐GGTCAACAAATCATAAAGATATTGG‐3'/HC02198 50‐TAAACTTCAGGGTGACCAAAAAATCA‐30) (Folmer et al., 1994), applied to those specimens preserved in >95% ethanol since collection or preserved in <95% ethanol but that were collected within one year of DNA extraction. For older or more degraded samples, that is, those preserved in <95% ethanol and that were more than 1 years old, the following primers were used to target shorter, overlapping segments of COI: Uni‐MinibarF1 (59‐TCCACTAATCACAARGATATTGGTAC‐39) and UniMinibarR1 (59‐GAAAATCATAATGAAGGCATGAGC39). These are primers designed for a short fragment at the 5’ terminus of the standard barcode region (Meusnier et al., 2008). Each PCR reaction was thermocycled at 94°C for 1 min; 5 cycles at 94°C for 40 s, 45°C for 40 s, 72°C for 1 min; 35 cycles at 94°C for 40 s, 51°C for 40 s, 72°C for 1 min; held at 72°C for 5 min, and stored at 4°C. Successful PCR reactions was evaluated using an Invitrogen 2% agarose E‐gel with an ethidium bromide stain and developed with UV, and if successful, was subsequently bi‐directionally sequenced using BigDye and an Applied Biosystems 3730XL DNA analyzer (Hajibabaei et al., 2005). All data associated with each specimen included in this study, including collection information, storing institution, ecological data, taxonomy, photographs, and COI sequences, are available in BOLD under the publicly accessible dataset titled “DS‐POLYCSS Nearctic Polycentropodidae (Trichoptera)” (Orfinger et al., 2021).

### 2.3 Sequence alignment, P‐distance calculation, and tree construction

A two‐tiered analytical approach was applied to datasets. Initially, a “pooled” dataset including all available sequences was used in executing all tree‐ and distance‐based analyses. Not only were initial associations gathered from the pooled analysis but species for which associations were not currently attainable were also recovered as targets for future association efforts. Following analysis of the pooled dataset, “filtered” datasets composed of only those species for which successful associations were recovered are used in a subsequent iteration of tree‐based and distance‐based analyses described below. The filtered datasets were delineated according to biogeographical assignment, with western Nearctic species assigned to the “western” dataset and eastern Nearctic species assigned to the “eastern” dataset following biogeographical patterns recognized in many Nearctic caddisfly taxa, including *Polycentropus* (e.g., Cooper & Morse, 1998; Hamilton, 1986; Lago & Harris, 1987; Prather & Morse, 2001; Trivette, 1969). The east–west geographic delineation follows a slight variation of the definition of Lago and Harris (1987) and Cooper and Morse (1998), where “eastern” refers to Manitoba and the US states adjacent to either side of the Mississippi River and eastward, and “western” pertains to the remaining Nearctic region.

A total of 262 sequences of males, females, pupae, and larvae representing 23 total species were included in the initial, pooled analysis. A total of 66 sequences of western species (representing 23 adults and 43 larvae) and 88 sequences of eastern species (representing 48 adults and 40 larvae) were used in the filtered datasets. All sequences used consisted of at least 325 base pairs. All data associated with specimens incorporated in the filtered datasets are accessible in Supplementary File 4 for the western fauna and Supplementary File 5 for the eastern fauna. COI sequences were aligned using default settings of MUSCLE (Edgar, 2004) in MEGA v. X 10.1.0 (Kumar et al., 2018) for each western and eastern taxon. The alignments were checked manually to avoid stop codons, indels, and amino acid translation frame shifts. Pairwise divergence distances (p‐distances) within‐ and between‐species divergences of COI nucleotides were calculated in MEGA v. X 10.1.0 (Kumar et al., 2018) using the Kimura 2‐parameter evolution model (K2P) (Kimura, 1980) and pairwise deletion of missing sites. P‐distance describes the proportion of nucleotide sites at which sequences being compared are different and is obtained by dividing the number of nucleotide differences by the total number of nucleotides compared. Lower pairwise distances are an indication of fewer changes in the nucleotide, with lower p‐distance values expected intraspecifically than interspecifically, termed the barcoding gap (Meyer & Paulay, 2005).

Unrooted neighbor‐joining (NJ) trees of all available haplotypes, with pairwise deletion of missing sites and K2P distances (Kimura, 1980), were constructed in MEGA v. X 10.1.0 (Tamura et al., 2007). Branch support was calculated using 1,000 bootstrap replicates.

Maximum likelihood (ML) analysis was performed to evaluate support further for the monophyletic groupings of species using a model‐based method. The optimal substitution model was identified using ModelTest‐NG v0.1.7 (Darriba et al., 2020) and assessed using AIC, AICc, and BIC criteria. The partitioning scheme was identified and implemented using RAxML version 8 (Stamatakis, 2014) via raxmlGUI 2.0.0 (Edler et al., 2021). Unrooted ML trees were inferred with a TIM2+G4 model using RAxML version 8 (Stamatakis, 2014) via raxmlGUI 2.0.0 (Edler et al., 2021). Bootstrap support was calculated from 1000 replicates. Resulting trees were visualized and annotated using the Interactive Tree Of Life (iTOL) v5 (Letunic & Bork, 2021) and Adobe Illustrator^®^ version 24.3. Adobe Illustrator version 24.3 was used to make final cosmetic edits, without altering branch lengths, bootstrap values, and topologies.

### 2.4 Larval–adult and male–female association

The molecular association of larval and female specimens follows criteria proposed by Zhou et al. (2007) and employed by Ruiter et al. (2013) based on a phylogenetic species conceptual approach. Briefly, when the sequence of an individual of an unknown species is identical to that of a confirmed male of a species (i.e., pairwise p‐distance is zero), is nested among near‐identical COI sequences of males of a species, or is nested in a monophyletic group of specimens of a given species on both trees, the corresponding species name was applied to the unknown female or larval individual.

## 3 RESULTS

### 3.1 Tree‐based associations

#### 3.1.1 Pooled Fauna

**FIGURE 1:**
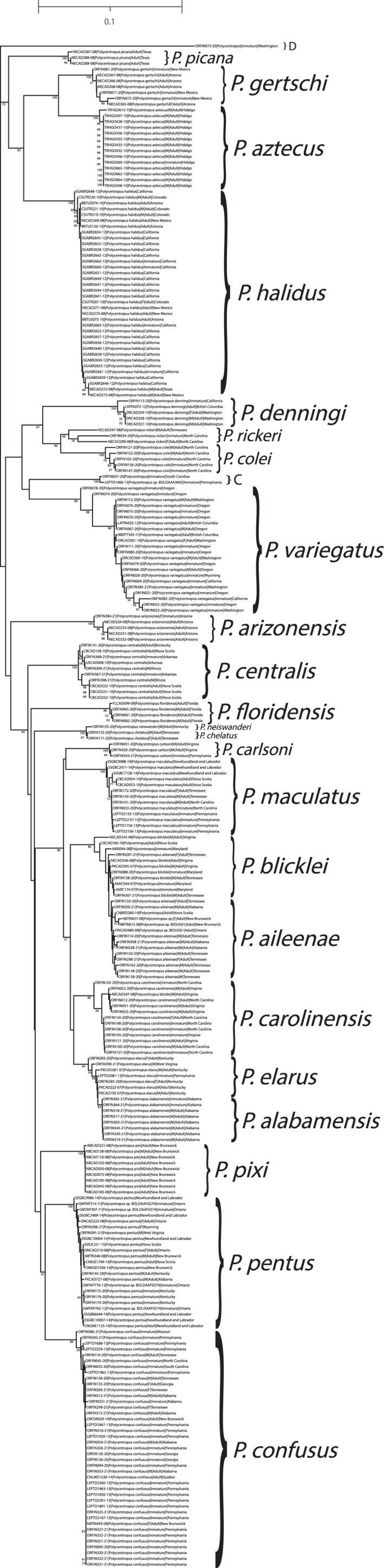
Initial neighbor‐joining tree for pooled mtCOI barcoding sequence data. Only bootstrap values ≥50% are shown. Specimen labels at branch tips include taxon, BOLD Sample ID, sex (if adult and available), and life stage. Scale bar indicates genetic distance

**FIGURE 2:**
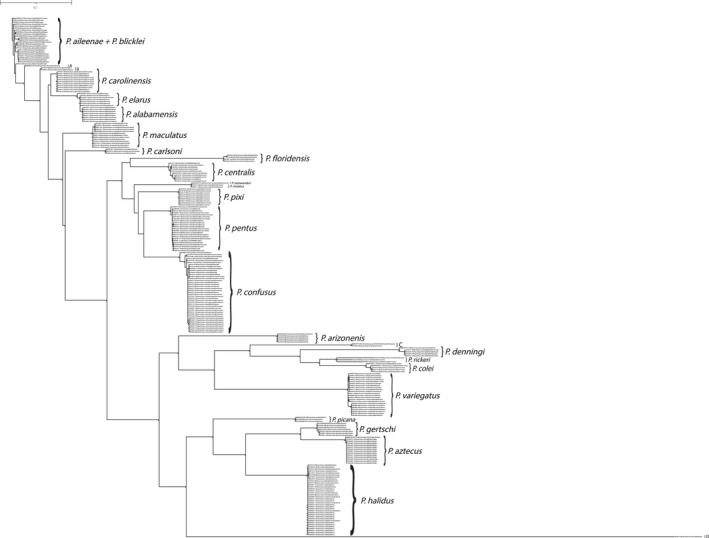
Initial maximum likelihood tree for pooled mtCOI barcoding sequence data. Only bootstrap values ≥50% are shown. Specimen labels at branch tips include taxon, BOLD Sample ID, sex (if adult and available), and life stage. Scale bar indicates substitutions per site

A total of 14 novel larval‐male associations and two male–female associations were indicated in the pooled analysis. Both the neighbor‐joining tree (Figure 1) and maximum likelihood tree (Figure 2) yielded species‐level monophyletic groupings with strong bootstrap support with two notable exceptions. While each species formed a monophyletic group with strong statistical support, *P. alabamensis* Hamilton et al., 1990 was nested among *P. elarus* Ross, 1944 sequences in both NJ and ML trees. Similarly, *P. aileenae* Orfinger & Moulton, 2021 was found to be nested within *P. blicklei* Ross & Yamamoto, 1965 in the NJ tree with low (<50) bootstrap support while the two species were formed a single, more admixed clade (*P. aileenae* + *P. blicklei*) with low (<50) bootstrap support in the ML tree.

The identities of four lineages were equivocal. These were termed clades ‘A’, ‘B’, ‘C’, and ‘D’. Clades B and C contained two specimens each, while A and D were each represented by a single unplaced specimen. Clade A occurred only in the ML tree and consisted of a male specimen (NECAD245‐08) identified as *P. blicklei* and sister to the *P. aileenae* + *P*. *blicklei* clade. This specimen is recovered in the NJ tree as a member of the *P. blicklei* clade. Clade A is sister to Clade B, which also occurs only in the ML tree. Clade B consists of two males identified as *P. blicklei* and *P. carolinensis* Banks, 1905 (specimens NECAD247‐08 and ORFIN052‐20, respectively). Both of these specimens are recovered as members of the *P. carolinensis* clade with strong support in the NJ tree. Clade C was recovered in both trees and is represented by two unassociated larval specimens, ORFIN047‐20 and LEPTO1466‐13, collected in South Carolina and Pennsylvania, respectively. Finally, Clade D occurs in each tree and is represented by a single, unassociated larva (ORFIN073‐20).

#### 3.1.2 Western Fauna

**FIGURE 3:**
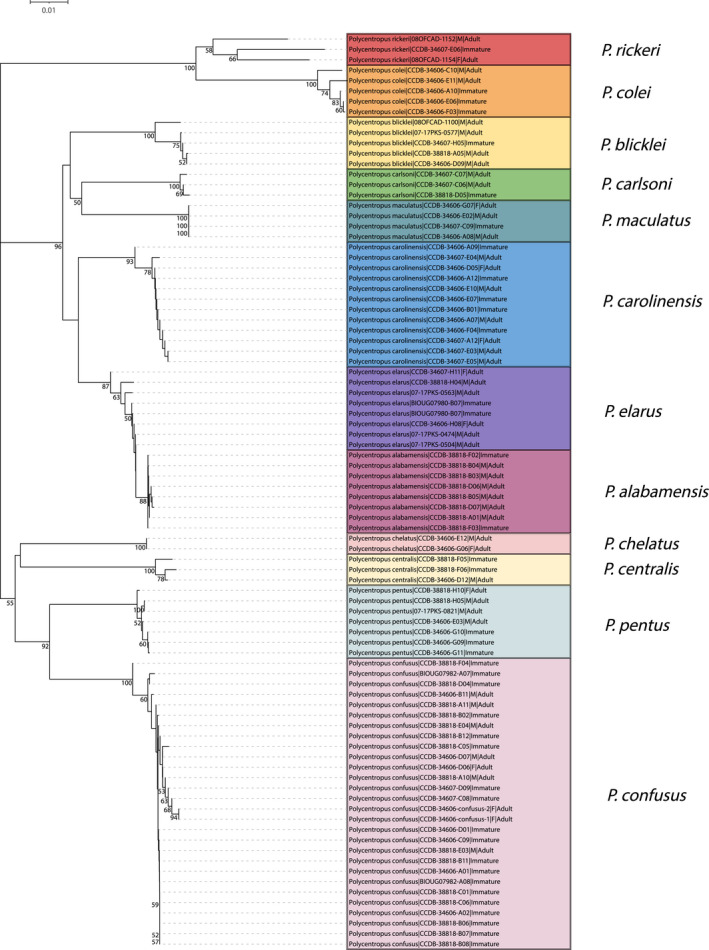
Neighbor‐joining tree for mtCOI barcoding sequence data of western taxa yielding successful associations. Only bootstrap values ≥50% are shown. Specimen labels at branch tips include taxon, BOLD Sample ID, sex (if adult and available), and life stage. Scale bar indicates genetic distance

**FIGURE 4:**
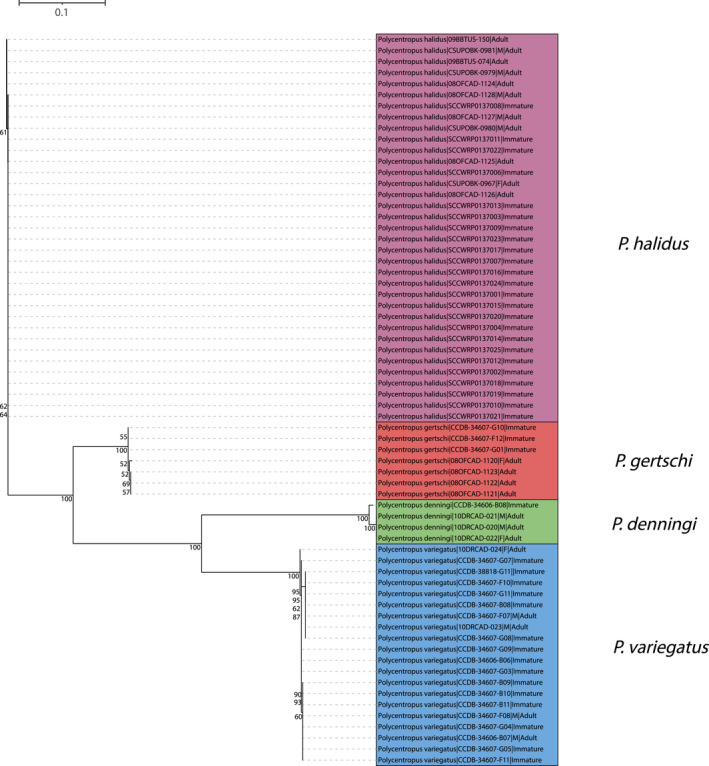
Neighbor‐joining tree for mtCOI barcoding sequence data of eastern taxa yielding successful associations. Only bootstrap values ≥50% are shown. Specimen labels at branch tips include taxon, BOLD Sample ID, sex (if adult and available), and life stage. Scale bar indicates genetic distance

In total, larvae of four of the seven western species were newly associated with confirmed adults, namely *Polycentropus denningi*, *Polycentropus gertschi* Denning, 1950, *Polycentropus halidus* Milne, 1936, and *Polycentropus variegatus*. Both the neighbor‐joining tree (Figure 3) and maximum likelihood tree (Figure 4) yielded species‐level monophyletic groups with strong bootstrap support.

#### 3.1.3 Eastern Fauna

In total, larvae of 10 of the 23 eastern species were newly associated with confirmed adults, namely *P. alabamensis*, *P. blicklei*, *P. carlsoni* Morse, 1971, *P. carolinensis*, *P. colei* Ross, 1941, *P. confusus* Hagen & Uhler, 1861, *P. elarus*, *P. maculatus* Banks, 1908, *P. pentus* Ross, 1941, and *P. rickeri* Yamamoto, 1966. A notable molecular association confirmed the identity of the previously described *Polycentropus centralis* Banks, 1914, which was known from a single, geographically associated specimen (Ross, 1944). Two novel female associations were also achieved for *P. carolinensis* and *P. chelatus* Ross & Yamamoto, 1965.

**FIGURE 5:**
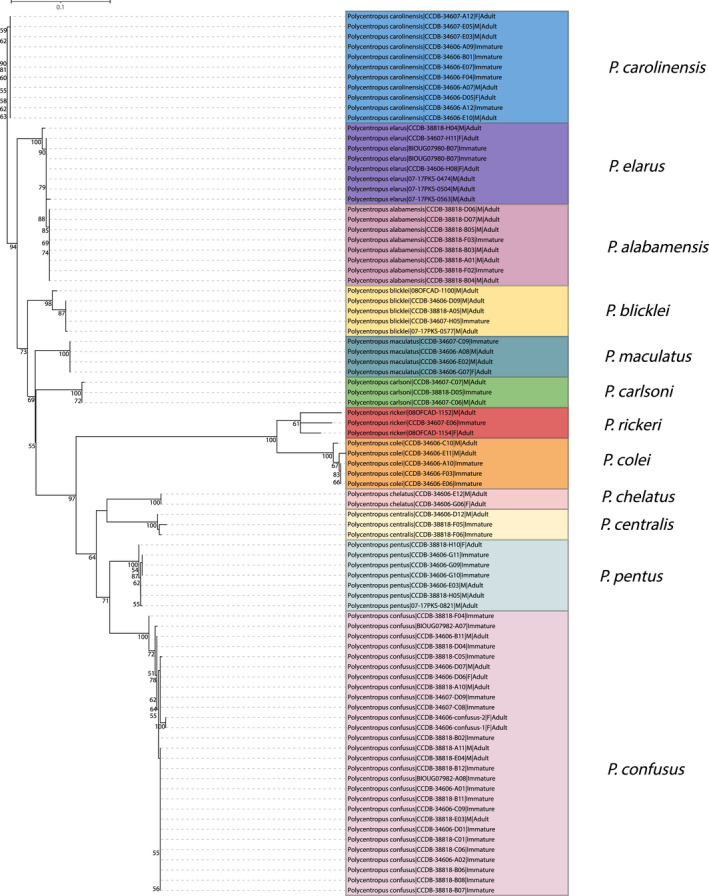
Maximum likelihood tree for mtCOI barcoding sequence data of western taxa yielding successful associations. Only bootstrap values ≥50% are shown. Specimen labels at branch tips include taxon, BOLD Sample ID, sex (if adult and available), and life stage. Scale bar indicates substitutions per site

**FIGURE 6:**
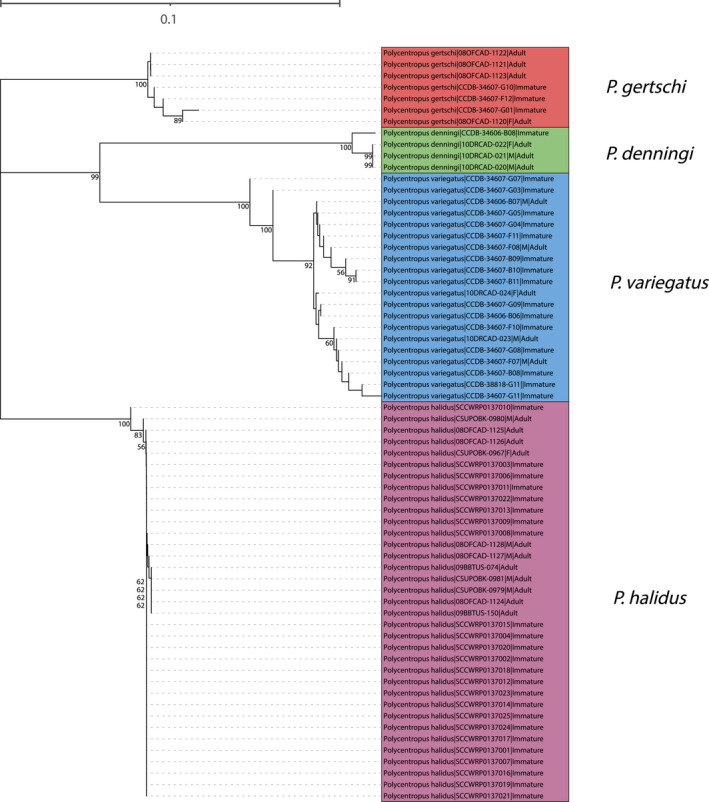
Maximum likelihood tree for mtCOI barcoding sequence data of eastern taxa yielding successful associations. Only bootstrap values ≥50% are shown. Specimen labels at branch tips include taxon, BOLD Sample ID, sex (if adult and available), and life stage. Scale bar indicates substitutions per site

Both the neighbor‐joining tree (Figure 5) and maximum likelihood tree (Figure 6) yielded species‐level monophyletic groupings with strong bootstrap support. While forming a monophyletic group, the *P. alabamensis* clade is nested within the *P. elarus* clade with high bootstrap support in each analysis.

### 3.2 Pairwise divergence distances

#### 3.2.1 Pooled Fauna

Summary p‐distance data of the pooled fauna are presented in Table 1. Maximum intraspecific p‐distance values were generally less than minimum interspecific values, indicating the existence of barcoding gaps. Notable exceptions reflect the ambiguous relationships recovered in the pooled NJ and ML trees. *Polycentropus aileenae* and *P. blicklei* exhibit a minimum distance of 0 and the maximum distance of 0.09. Similarly, *P. blicklei* and *P. carolinensis* exhibit a minimum distance of 0 and the maximum distance of 0.06, reflecting the ambiguity of clade B. Also notable is the very small p‐distance range between *P. alabamensis* and *P. elarus*, spanning a minimum of 0 and a maximum of only 0.01. The complete pairwise p‐distance comparison for pooled fauna is available in Supplementary File 1.

**TABLE 1:** Ranges of pairwise divergence (p‐distance) among taxa for the pooled mtCOI dataset

Species	*Polycentropus aileenae*		*Polycentropus alabamensis*		*Polycentropus arizonensis*		*Polycentropus aztecus*		*Polycentropus blicklei*		*Polycentropus carlsoni*		*Polycentropus carolinensis*		*Polycentropus centralis*		*Polycentropus chelatus*		*Polycentropus colei*		*Polycentropus confusus*	
	Min	Max	Min	Max	Min	Max	Min	Max	Min	Max	Min	Max	Min	Max	Min	Max	Min	Max	Min	Max	Min	Max
*P. aileenae*	0	**0.07**	0.05	0.1	0.12	0.18	0.14	0.18	0	0.09	0.06	0.13	0.04	0.13	0.09	0.15	0.09	0.14	0.12	0.16	0.07	0.14
*P. alabamensis*			0	**0**	0.11	0.14	0.14	0.15	0.04	0.05	0.07	0.07	0.03	0.04	0.09	0.1	0.09	0.1	0.13	0.16	0.06	0.08
*P. arizonensis*					0	**0.01**	0.14	0.16	0.11	0.15	0.09	0.15	0.1	0.15	0.11	0.13	0.1	0.14	0.14	0.17	0.09	0.16
*P. aztecus*							0	**0.01**	0.12	0.14	0.12	0.14	0.13	0.14	0.14	0.14	0.13	0.14	0.16	0.17	0.11	0.18
*P. blicklei*									0	**0.02**	0.05	0.08	0	0.06	0.07	0.1	0.07	0.09	0.12	0.14	0.07	0.13
*P. carlsoni*											0	**0**	0.03	0.06	0.09	0.1	0.09	0.1	0.12	0.13	0.04	0.1
*P. carolinensis*													0	**0**	0.08	0.11	0.07	0.11	0.13	0.16	0.07	0.12
*P. centralis*															0	**0.01**	0.07	0.08	0.12	0.15	0.06	0.12
*P. chelatus*																	0	**0**	0.12	0.13	0.06	0.12
*P. colei*																			0	**0.02**	0.12	0.17
*P. confusus*																					0	**0.01**
*P. denningi*																						
*P. elarus*																						
*P. floridensis*																						
*P. halidus*																						
*P. gertschi*																						
*P. maculatus*																						
*P. neiswanderi*																						
*P. pentus*																						
*P. picana*																						
*P. pixi*																						
*P. rickeri*																						
*P. variegatus*																						

Species	*Polycentropus denningi*		*Polycentropus elarus*		*Polycentropus floridensis*		*Polycentropus gertschi*		*Polycentropus halidus*		*Polycentropus maculatus*		*Polycentropus neiswanderi*		*Polycentropus pentus*		*Polycentropus picana*		*Polycentropus pixi*		*Polycentropus rickeri*		*Polycentropus variegatus*	
	Min	Max	Min	Max	Min	Max	Min	Max	Min	Max	Min	Max	Min	Max	Min	Max	Min	Max	Min	Max	Min	Max	Min	Max
*P. aileenae*	0.16	0.19	0.04	0.1	0.13	0.19	0.13	0.19	0.12	0.17	0.06	0.12	0.09	0.14	0.04	0.14	0.12	0.17	0.09	0.16	0.12	0.16	0.08	0.19
*P. alabamensis*	0.14	0.16	0	0.01	0.12	0.13	0.13	0.14	0.11	0.13	0.05	0.06	0.08	0.08	0.06	0.09	0.11	0.11	0.09	0.1	0.12	0.13	0.07	0.17
*P. arizonensis*	0.17	0.19	0.1	0.14	0.14	0.18	0.13	0.17	0.11	0.15	0.12	0.15	0.11	0.14	10	0.15	0.11	0.13	0.09	0.14	0.1	0.17	0.09	0.17
*P. aztecus*	0.18	0.19	0.12	0.15	0.16	0.17	0.06	0.07	0.1	0.11	0.15	0.16	0.14	0.15	0.11	0.15	0.1	0.11	0.14	0.16	0.14	0.18	0.12	0.18
*P. blicklei*	0.15	0.16	0.03	0.06	0.11	0.15	0.12	0.15	0.11	0.13	0.05	0.08	0.07	0.09	0.06	0.09	0.12	0.13	0.08	0.1	0.12	0.16	0.08	0.18
*P. carlsoni*	0.14	0.16	0.05	0.07	0.1	0.12	0.14	0.16	0.09	0.12	0.05	0.06	0.09	0.1	0.06	0.09	0.1	0.12	0.08	0.09	0.12	0.14	0.09	0.2
*P. carolinensis*	0.11	0.15	0	0.04	0.12	0.13	0.12	0.14	0.1	0.14	0.04	0.05	0.08	0.11	0.06	0.1	0.09	0.13	0.08	0.11	0.11	0.14	0.07	0.18
*P. centralis*	0.12	0.17	0.06	0.1	0.1	0.11	0.12	0.17	0.12	0.17	0.09	0.11	0.07	0.08	0.07	0.09	0.11	0.12	0.07	0.1	0.12	0.15	0.06	0.19
*P. chelatus*	0.15	0.17	0.06	0.09	0.09	0.12	0.14	0.16	0.12	0.14	0.09	0.09	0.01	0.01	0.06	0.07	0.12	0.12	0.07	0.07	0.11	0.13	0.07	0.19
*P. colei*	0.11	0.13	0.11	0.15	0.16	0.18	0.14	0.17	0.13	0.17	0.13	0.15	0.13	0.14	0.13	0.16	0.15	0.16	0.13	0.15	0.06	0.07	0.1	0.15
*P. confusus*	0.13	0.18	0.05	0.1	0.08	0.15	0.11	0.19	0.07	0.16	0.08	0.1	0.07	0.12	0.04	0.11	0.08	0.14	0.05	0.13	0.1	0.15	0.05	0.18
*P. denningi*	0	**0.01**	0.14	0.16	0.17	0.18	0.16	0.21	0.13	0.18	0.15	0.16	0.16	0.17	0.11	0.17	0.17	0.17	0.14	0.15	0.1	0.12	0.12	0.17
*P. elarus*			0	**0.01**	0.1	0.14	0.11	0.14	0.1	0.13	0.05	0.06	0.07	0.09	0.06	0.09	0.1	0.12	0.08	0.1	0.1	0.14	0.06	0.17
*P. floridensis*					0	**0.01**	0.17	0.19	0.15	0.17	0.12	0.14	0.1	0.12	0.09	0.11	0.14	0.15	0.1	0.12	0.13	0.15	0.09	0.18
*P. halidus*							0.07	0.11	0	**0**	0.12	0.14	0.12	0.15	0.1	0.13	0.09	0.12	0.12	0.15	0.11	0.17	0.09	0.19
*P. gertschi*							0	**0.01**	0.08	0.11	0.14	0.16	0.14	0.17	0.12	0.17	0.11	0.13	0.14	0.17	0.16	0.18	0.1	0.19
*P. maculatus*											0	**0**	0.09	0.11	0.08	0.11	0.11	0.13	0.1	0.11	0.12	0.14	0.09	0.19
*P. neiswanderi*													N/A	N/A	0.07	0.08	0.12	0.12	0.07	0.07	0.11	0.14	0.08	0.19
*P. pentus*															0	**0**	0.08	0.13	0.05	0.06	0.12	0.15	0.06	0.2
*P. picana*																	0	**0**	0.11	0.13	0.14	0.16	0.09	0.15
*P. pixi*																			0	**0.01**	0.12	0.14	0.07	0.2
*P. rickeri*																					0.04	**0.05**	0.13	0.16
*P. variegatus*																							0	**0.02**

Note

Values are rounded to two decimal places. Maximum intraspecific values are displayed in bold.

#### 3.2.2 Western Fauna

The complete pairwise p‐distance matrix for western fauna is available in Supplementary File 2. P‐distance analysis corroborates tree‐based analyses with instances of pairwise p‐distances between sequences of adults and immature specimens being zero. For example, the larval specimen of *P. halidus* with BOLD specimen ID SCCWRP0137008 shared an identical sequence with the male with BOLD specimen ID 09BBTUS‐074, indicating that they are the same species. There were no instances of high p‐distance values between congeners including immature and female specimens, with all intraspecific values ≤0.01.

Summary p‐distance data of the western fauna are presented in Table 2. Maximum intraspecific p‐distance values were always far less than minimum interspecific values, indicating the existence of barcoding gaps. Considering the inclusion of larvae in pairwise p‐distance comparison, the presence of a barcode gap supports the specific assignments of larvae obtained from the phylogenetic analyses.

**TABLE 2:** Ranges of pairwise divergence (p‐distance) among taxa for the western mtCOI dataset for which associations were accomplished

Species	*Polycentropus denningi*		*Polycentropus halidus*		*Polycentropus gertschi*		*Polycentropus variegatus*	
	Min	Max	Min	Max	Min	Max	Min	Max
*P. denningi*	0	**0.01**	0.13	0.16	0.16	0.19	0.12	0.15
*P. halidus*			0	**0**	0.07	0.11	0.1	0.17
*P. gertschi*					0	**0.01**	0.1	0.15
*P. variegatus*							0	**0.01**

Note

Values are rounded to two decimal places. Maximum intraspecific values are displayed in bold.

#### 3.2.3 Eastern Fauna

The full pairwise p‐distance matrix for eastern fauna is available in Supplementary File 2. As with the western faunal analysis, p‐distance analysis corroborates tree‐based analyses with instances of pairwise p‐distances between sequences of adults and immature specimens being zero. For example, the female *P. carolinensis* with BOLD specimen ID CCDB‐34606‐D05 had an identical COI sequence as the male with BOLD specimen ID CCDB‐34606‐E10 and the larva with BOLD specimen ID CCDB‐34606‐E07. Similarly, the female *P. chelatus* with BOLD specimen ID CCDB‐34606‐G06 produced a COI sequence identical to the male with BOLD specimen ID CCDB‐34606‐E12.

Summary p‐distance data of the eastern fauna are presented in Table 3. Maximum intraspecific p‐distance values were generally far less than minimum interspecific values, indicating the existence of barcoding gaps. Considering the inclusion of larvae in pairwise p‐distance comparison, the presence of a barcode gap supports the specific assignments of larvae obtained from the phylogenetic analyses.

**TABLE 3:** Ranges of pairwise divergence (p‐distance) among taxa for the eastern mtCOI dataset for which associations were accomplished

Species	*Polycentropus alabamensis*		*Polycentropus blicklei*		*Polycentropus carlsoni*		*Polycentropus carolinensis*		*Polycentropus centralis*		*Polycentropus chelatus*		*Polycentropus colei*		*Polycentropus confusus*		*Polycentropus elarus*		*Polycentropus maculatus*		*Polycentropus pentus*		*Polycentropus rickeri*	
	Min	Max	Min	Max	Min	Max	Min	Max	Min	Max	Min	Max	Min	Max	Min	Max	Min	Max	Min	Max	Min	Max	Min	Max
*P. alabamensis*	0	**0**	0.04	0.05	0.07	0.07	0.03	0.04	0.09	0.09	0.09	0.1	0.13	0.14	0.08	0.08	0	0.01	0.05	0.06	0.08	0.08	0.12	0.13
*P. blicklei*			0	**0.03**	0.05	0.08	0.03	0.06	0.07	0.09	0.07	0.09	0.12	0.14	0.08	0.1	0.04	0.05	0.05	0.06	0.07	0.09	0.12	0.16
*P. carlsoni*					0	**0**	0.05	0.06	0.09	0.1	0.09	0.1	0.12	0.13	0.08	0.1	0.05	0.07	0.05	0.06	0.07	0.09	0.12	0.13
*P. carolinensis*							0	**0**	0.08	0.11	0.07	0.11	0.13	0.14	0.07	0.1	0	0.04	0.5	0.05	0.06	0.09	0.11	0.13
*P. centralis*									0	**0.01**	0.07	0.08	0.12	0.13	0.07	0.09	0.06	0.09	0.09	0.09	0.07	0.08	0.12	0.14
*P. chelatus*											0	**0**	0.12	0.13	0.07	0.08	0.07	0.09	0.09	0.09	0.06	0.07	0.11	0.13
*P. colei*													0	**0.02**	0.13	0.14	0.11	0.14	0.13	0.13	0.13	0.15	0.06	0.07
*P. confusus*															0	**0.01**	0.06	0.08	0.08	0.1	0.04	0.06	0.1	0.14
*P. elarus*																	0	**0.01**	0.05	0.05	0.06	0.08	0.1	0.14
*P. maculatus*																			0	**0**	0.09	0.1	0.12	0.12
*P. pentus*																					0	**0**	0.12	0.14
*P. rickeri*																							0.04	**0.05**

Note

Values are rounded to two decimal places. Maximum intraspecific values are displayed in bold.

Two exceptions from these observations exist in *P. alabamensis* and *P. rickeri*. Specimens of *P. alabamensis* do not demonstrate a barcode gap with respect to *P. elarus*. This is mirrored in the phylogenies, with the *P. alabamensis* clade nested within the *P. elarus* clade. Meanwhile, *P. rickeri* specimens present high intraspecific pairwise p‐distances, with a minimum of 0.04 and a maximum of 0.05 based on only three sequences.

### 3.3 Morphological corroboration

Like males, female caddisflies are generally identified to species according to morphological aspects of the genitalia. For example, Ross (1944) provided a key to females of the *Polycentropus sensu lato* (i.e., *Holocentropus* McLachlan, 1878, *Plectrocnemia* Stephens, 1836, and *Polycentropus*) based largely on ventral views of cleared genitalia. Initial examination of the two females newly associated here suggest that the ventral plates and internal parts of the gonopods enable separation from other members of the *Polycentropus confusus* Species Group to which they belong.

**FIGURE 7:**
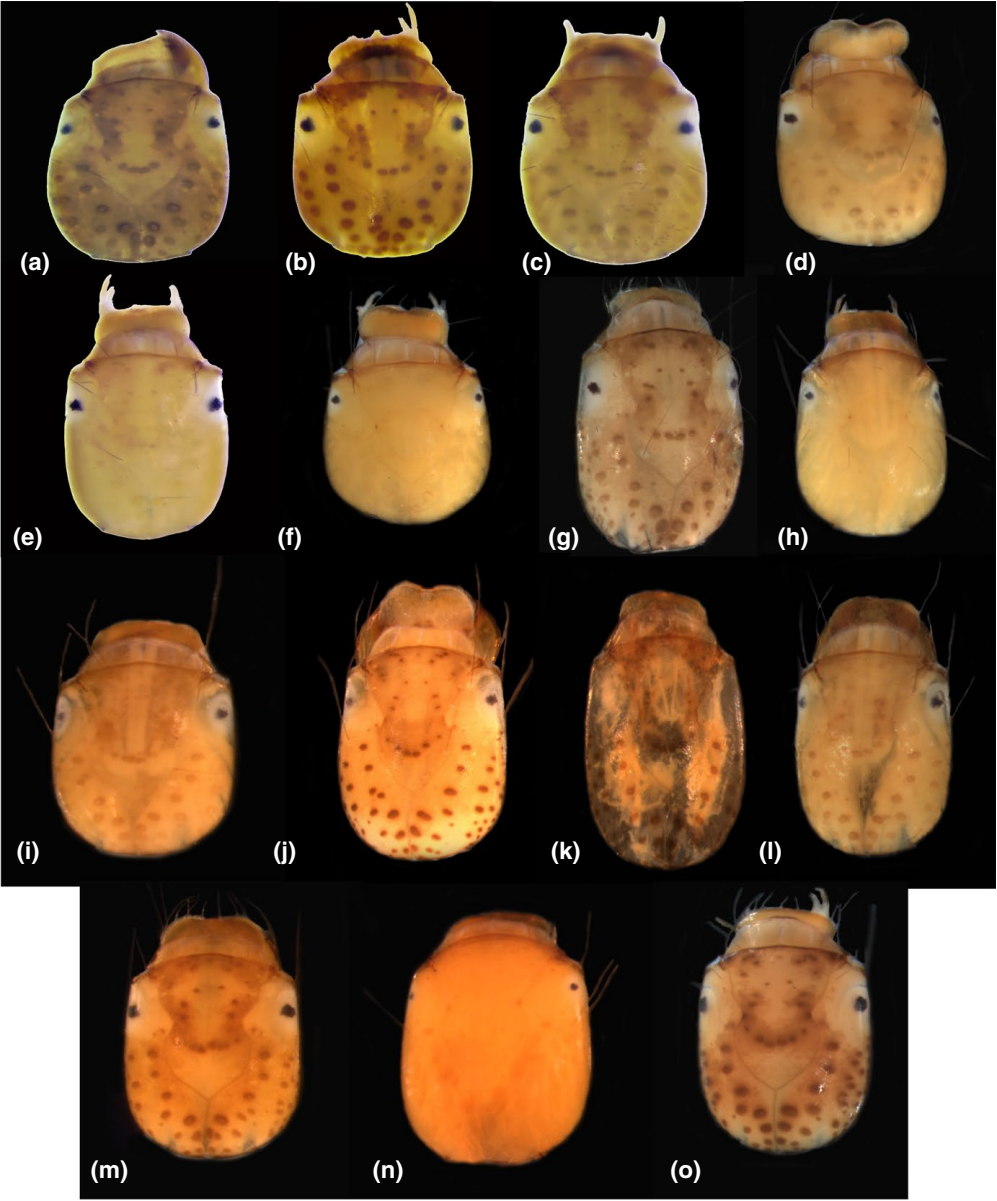
Head capsules of associated Nearctic species of *Polycentropus* Curtis, 1835 larva, dorsal views. (a) *P. alabamensis* Hamilton et al., 1990; (b) *P. blicklei* Ross & Yamamoto, 1965; (c) *P. carlsoni* Morse, 1971; (d) *P. carolinensis* Banks, 1905; (e) *P. centralis* Banks, 1914; (f) *P. colei* Ross, 1941; (g) *P. confusus* Hagen & Uhler, 1861; (h) *P. denningi* Smith, 1962, (i) *P. elarus* Ross, 1944; (j) *P. gertschi* Denning, 1950; (k) *P. halidus* Milne, 1936; (l) *P. maculatus* Banks, 1908; (m) *P. pentus* Ross, 1941; (n) *P. rickeri* Yamamoto, 1966; (o) *P. variegatus* Banks, 1900. Note that the head capsule of the *P. halidus* specimen underwent lysis during DNA extraction, removing soft tissue but maintaining the sclerotized head capsule before it was photographed

The newly associated larvae reported here also appear separable by various aspects of their morphology. For example, Figure 7 illustrates the apparently consistent interspecific differences in head coloration, roundness, pigment banding, and muscle scar patterning. The degree to which anal claws are curved also appears intraspecifically consistently similar and interspecifically consistently different, with some demonstrating sharply curved anal claws and others possessing gradually curved anal claws.

## 4 DISCUSSION

While the work presented here is not intended to infer phylogenetic relationships, some interesting observations arose that merit brief discussion. First, the nested position of *P. alabamensis* relative to the *P. elarus* clade recovered in neighbor‐joining and maximum likelihood phylogenies suggests a very close relationship between the species, or even that *P. alabamensis* could be a synonym of *P. elarus*. Genetic distances presented in Tables 1 and 2 suggest the latter conclusion. Still, synonymy is unlikely given the distinct differences readily observable in the male genitalia of each species [e.g., see figures 1A–1F by Hamilton et al. (1990) and figures 245A–245C of *P. elarus* by Ross (1944)]. Instead, it seems more plausible that these two species are closely related sister taxa. A robust phylogenetic study of the *Polycentropus confusus* Species Group, of which these two species belong, will help to resolve this question. Such a study is currently under way, incorporating multiple loci and morphology. So, too, will comparative descriptions of the now‐identified larval stages of each species shed light on their relationships.

A second notable observation is the high intraspecific genetic distance observed in the *P. rickeri* (Tables 1 and 2). From sequences of only two male specimens and one immature specimen, pairwise intraspecific distances spanned 0.04 to 0.05, or 4% to 5%. These data more than twice exceed the 2% COI distance threshold often employed to delineate species (Hebert et al., 2003; Meyer & Paulay, 2005; Rivera & Currie, 2009; Sweeney et al., 2011; White et al., 2014). The high values suggest the existence of cryptic species. This species has been reported from seven eastern U.S. states (Rasmussen & Morse, 2020). Future sampling and generation of additional molecular data coupled with morphological study of exemplars from throughout its range should be performed to test for potential cryptic species. Such an in‐depth investigation will be necessary to verify the association proposed here based on the monophyly of the *P. rickeri* clade in the inferred trees.

If combined with investigations of *P. barri* Ross & Yamamoto, 1965 and *P. colei*, a phylogenetic study will also evaluate the relationships of the *P. colei* Species Group, which consists of *P. barri*, *P. colei*, and *P. rickeri*. Among the eastern NJ and ML tree topologies, *P. colei* and *P. rickeri* are recovered as sister taxa with high statistical support. This relationship makes sense within the current classification scheme. While no phylogeny exists yet for this Species Group, the relationship observed in the trees produced here suggests the Species Group is monophyletic. Similarly, among the western taxa, *P. denningi* and *P. variegatus* were recovered as sister taxa with high statistical support within both NJ and ML trees. This suggests close relatedness of these taxa currently unplaced in any Species Group, despite the distinct genitalia of their males (e.g., see figures by Armitage & Hamilton, 1990).

The relationship between *P. aileenae* and *P. blicklei* is similar to that of *P. alabamensis* and *P. elarus*, although with much lower statistical support in separating the former pair. This is not surprising considering a close sister relationship was hypothesized by Orfinger and Moulton (2021) given the morphological similarity of both of sexes the two species. In such cases of closely related Species Groups and sister species, COI is often insufficient by itself in resolving these relationships. For example, while COI was unable to separate the five Finnish members of the *Apatania zonella* (Zetterstedt, 1840) Species Group (Apataniidae), morphology and more than 2 million bp of double digest RAD sequencing (ddRAD‐seq) sequence data supported the species hypotheses (Salokannel et al., 2021). A robust phylogeny combining morphology, COI DNA barcoding data, and additional molecular data will be needed to better refine our understanding of the relationship between these two taxa.

Several specimens merit further examination to resolve their specific identity. While ambiguous in the pooled ML tree, the male specimen NECAD247‐08 is recovered with strong support as a member of the *P. carolinensis* clade in the pooled NJ tree. The first author examined the other male specimen of clade B (ORFIN052‐20) which agrees with *P. carolinensis*. Therefore, it is likely that specimen NECAD247‐08 was misidentified as *P. blicklei* and is in fact *P. carolinensis*, although examination of that specimen is needed to confirm.

The unassociated larvae of clade C are interesting targets for additional scrutiny. Specimen ORFIN047‐20 from South Carolina does not completely agree with any of the associated larvae. Examination of the other specimen from Pennsylvania, LEPTO1466‐13, is required to confirm that these two unassociated larvae represent the same species. Unfortunately, this specimen is currently unavailable for examination due to the ongoing COVID‐19 pandemic but will be examined in the future once available. Another larva of ambiguous identify is specimen AMII094‐08, which may represent *P. aileenae* or *P. blicklei*. Its examination and comparison to *P. blicklei* larvae will be helpful in understanding its identity. As with specimen LEPTO1466‐13, however, this specimen is housed in the same collection and currently unavailable. The final unassociated larva, ORFIN073‐20, comprises clade D and was collected in Washington state. According to the pooled ML and NJ trees, the specimen is closely allied to the *Polycentropus gertschi* Species Group, but is clearly distinct. This specimen is also subtly morphologically different from other associated larvae. It is possible that this specimen and the members of clade C represent undescribed species. Alternatively, high‐quality COI sequence exemplars may not yet be available for adults of the species, precluding molecular association. For example, unidentified members of clade C could represent *Polycentropus barri*, an eastern species and member of the *Polycentropus colei* Species Group along with *P. colei* and *P. rickeri*, for which no sequence data are available. Future adult sampling from near the collection localities of these specimens, coupled with additional COI sequencing to associate the adults, will be required to identify these larvae.

A single pupal specimen (ORFIN384‐21) was identified as *P. arizonensis*. Unfortunately, examination of this specimen revealed that the pupal casing was absent and larval sclerites were lost. Therefore, the specimen is not useful in associating the male and larva of the species via the metamorphotype method (Milne, 1938). Still, while pupal association was not a goal of this study, this is the first reported identification of the pupa of this species, enabling its future morphological study.

Previously, *Polycentropus centralis* was the only Nearctic *Polycentropus* species with an associated larva, based on a presumed geographic association (Ross, 1944). This association and description were based on a single specimen from Illinois and was confirmed with the molecular association of two additional specimens from Missouri in light of corroborating morphology to be described in a future publication.

In addition to *P. centralis*, the larvae of four western and 10 eastern species are newly associated, bringing the total number of identified Nearctic *Polycentropus* larvae to 15 of 30 species, or 50% of the known fauna. These novel associations pave the way for the morphological description and diagnoses of those species’ larvae. The noted morphological characters that appear useful in separating larvae of different species treated here agree with previously published morphological data used to separate polycentropodid larvae, for example, in the former USSR (Lepneva, 1964, 1970), England (Hickin, 1967), and central Europe (Waringer & Graf, 2011). It is likely that these characters, coupled with distinct eastern or western Nearctic geographic distributions, will enable the generation of diagnostic matrices and dichotomous keys to species for identification of the now‐associated larvae.

This study constitutes the initial step in this taxonomic process, which aims to culminate in identification tools useful for basic research and applied freshwater bioassessment strategies that utilize caddisfly larvae as sentinels of water quality (Behrens‐Chapuis et al., 2021; Resh et al., 1995; Sweeney et al., 2011; White et al., 2014). In addition to novel larval associations, the newly associated females of *P. carolinensis* and *P. chelatus* serve to provide material for their descriptions and diagnoses. While historically neglected, identifying female aquatic insects in biological surveys can greatly influence the number of recorded species in an area or at a given time (e.g., Ekrem et al., 2010). Now, females of 17 Nearctic *Polycentropus* species are associated, constituting 57% of the known fauna. In many cases, female associations also allow for recognition and descriptions of the eggs of given species as well (e.g., by Orfinger & Moulton, 2021).

Until the description and diagnoses of the associated larvae and females are complete, and perhaps beyond that point, the newly generated molecular data presented here can serve to identify unknown larvae. Molecular identification of aquatic macroinvertebrates has been increasingly used in concert with traditional morphological identification in freshwater bioassessment (Behrens‐Chapuis et al., 2021; Sweeney et al., 2011; White et al., 2014). The data on which the current analyses are based are publicly available and should serve as a reference library for the Nearctic *Polycentropus* (Orfinger et al., 2021).

Efforts by the authors will continue to attempt to associate additional Nearctic *Polycentropus* larvae, pupae, females, and males using both mtDNA barcoding and traditional methods such as the metamorphotype method (Milne, 1938). Additional sampling and sequencing of the now‐associated larvae and females will also be targeted to capture geographic haplotype and morphological variation. The ultimate goal is to associate and describe all life stages of the Nearctic Polycentropodidae. From there, the study of each species’ morphology and ecology will be tractable. This study represents a significant step in this direction.

## CONFLICT OF INTEREST

The authors have no competing interests to declare.

## AUTHOR CONTRIBUTIONS

**Alexander B. Orfinger:** Conceptualization (equal); Data curation (equal); Formal analysis (equal); Investigation (equal); Methodology (equal); Project administration (equal); Software (equal); Supervision (equal); Validation (equal); Visualization (equal); Writing – original draft (equal); Writing – review & editing (equal). **John C. Morse:** Conceptualization (equal); Resources (equal); Supervision (equal); Validation (equal); Writing – review & editing (equal). **Raymond L. Hix:** Funding acquisition (equal); Project administration (equal); Resources (equal); Validation (equal); Writing – review & editing (equal).

### OPEN RESEARCH BADGES

This article has been awarded Open Materials, Open Data Badges. All materials and data are publicly accessible via the Open Science Framework at [https://doi.org/10.5883/DS‐POLYCS].

## Supporting information

Supplementary Material

Supplementary Material

Supplementary Material

Supplementary Material

Supplementary Material

## Data Availability

All sequence data are publicly available in BOLD in a dataset titled “DS‐POLYCSS Nearctic Polycentropodidae (Trichoptera)”, accessible at http://www.boldsystems.org/index.php/Public_SearchTerms?query=DS‐POLYCSS and via DOI https://doi.org/10.5883/DS‐POLYCSS. The metadata were summarized by Orfinger et al. (2021) and all associated specimen data are available in Supplementary Files 4 and 5 for western and eastern specimens, respectively. Complete p‐distance matrices for pooled, western, and eastern COI data are available in Supplementary Files 1, 2, and 3, respectively.
