# Corrigendum

**DOI:** 10.1002/ece3.8967

**Published:** 2022-07-06

In the recent article by Orfinger et al. (2022), Figures 3, 4, 5, 6 were published with incorrect captions.

The figures with correct captions are shown below: 

**FIGURE 3:**
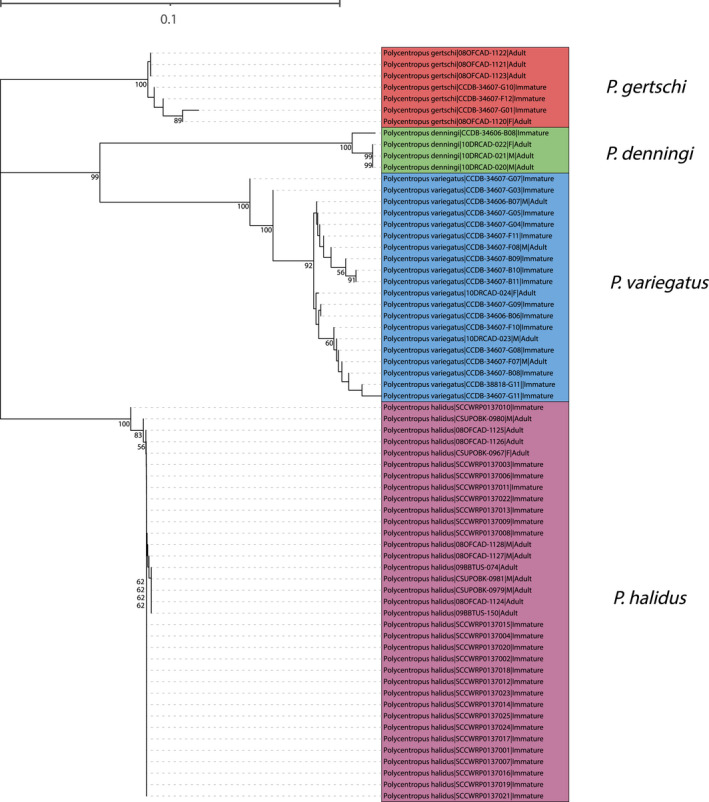
Neighbor‐joining tree for mtCOI barcoding sequence data of western taxa yielding successful associations. Only bootstrap values ≥50% are shown. Specimen labels at branch tips include taxon, BOLD Sample ID, sex (if adult and available), and life stage. Scale bar indicates genetic distance

**FIGURE 4:**
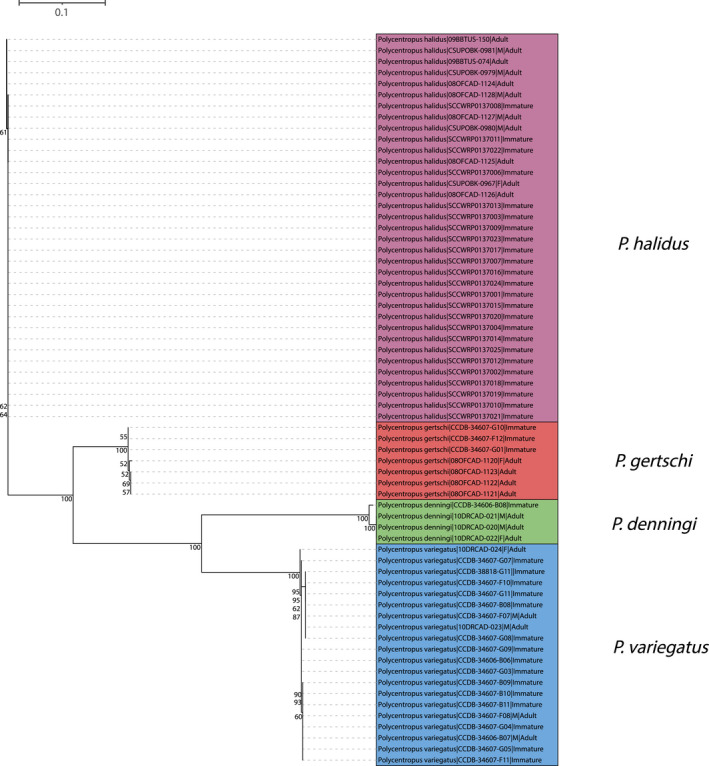
Maximum likelihood tree for mtCOI barcoding sequence data of western taxa yielding successful associations. Only bootstrap values ≥50% are shown. Specimen labels at branch tips include taxon, BOLD Sample ID, sex (if adult and available), and life stage. Scale bar indicates substitutions per site

**FIGURE 5:**
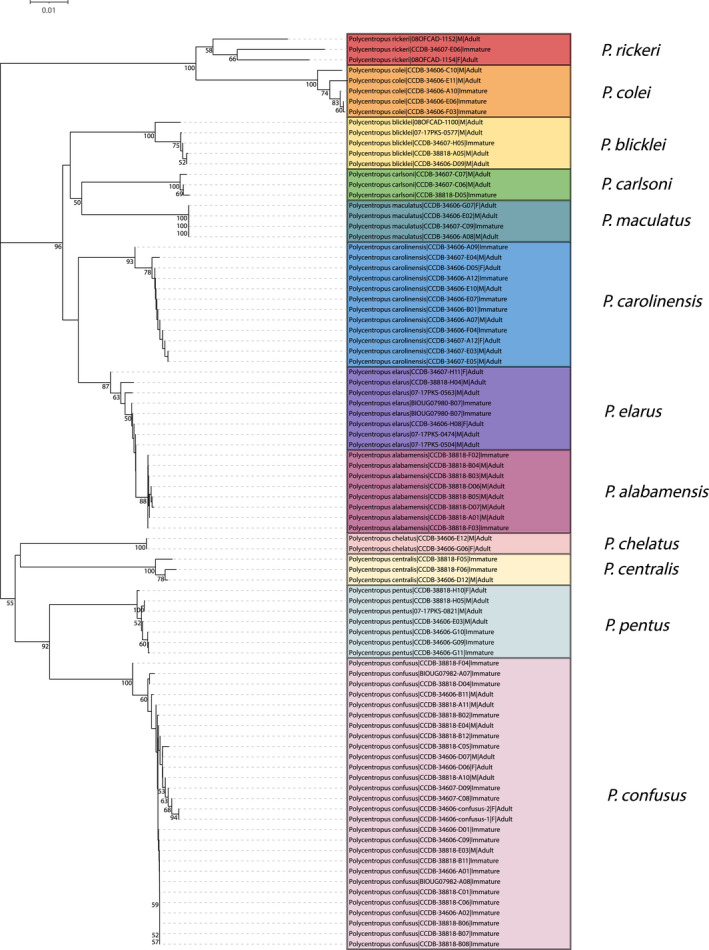
Neighbor‐joining tree for mtCOI barcoding sequence data of eastern taxa yielding successful associations. Only bootstrap values ≥50% are shown. Specimen labels at branch tips include taxon, BOLD Sample ID, sex (if adult and available), and life stage. Scale bar indicates genetic distance

**FIGURE 6:**
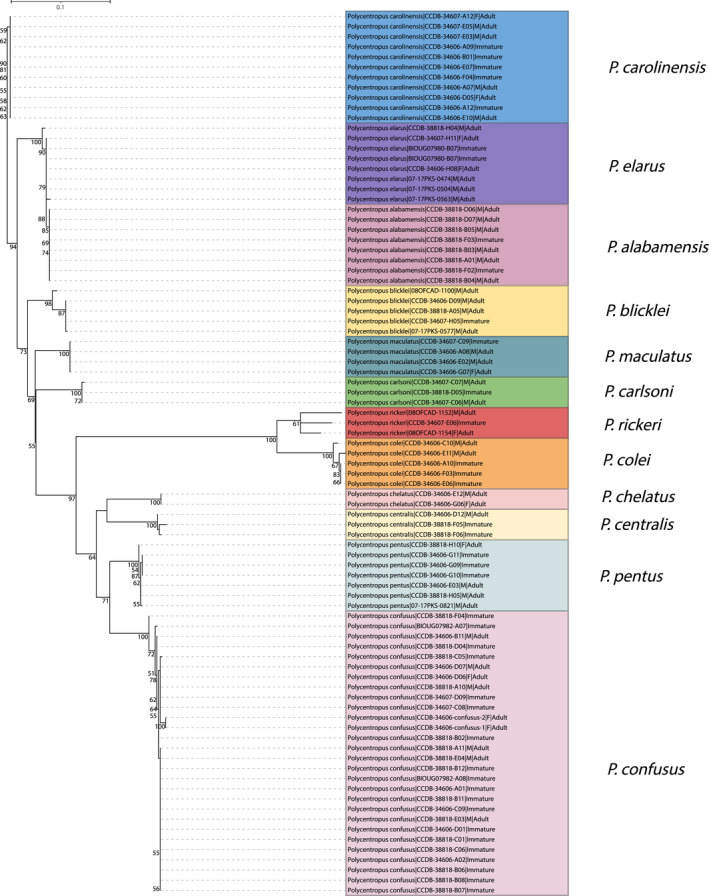
Maximum likelihood tree for mtCOI barcoding sequence data of eastern taxa yielding successful associations. Only bootstrap values ≥50% are shown. Specimen labels at branch tips include taxon, BOLD Sample ID, sex (if adult and available), and life stage. Scale bar indicates substitutions per site
